# Akt/mTOR mediated induction of bystander effect signaling in a nucleus independent manner in irradiated human lung adenocarcinoma epithelial cells

**DOI:** 10.18632/oncotarget.14931

**Published:** 2017-02-01

**Authors:** Lu Li, Lu Wang, Kevin M. Prise, K.N . Yu, Guodong Chen, Lianyun Chen, Yide Mei, Wei Han

**Affiliations:** ^1^ Anhui Province Key Laboratory of Medical Physics and Technology/Center of Medical Physics and Technology, Hefei Institutes of Physical Sciences, Chinese Academy of Sciences, Hefei, Anhui, China; ^2^ Centre for Cancer Research & Cell Biology, Queen's University, Belfast, UK; ^3^ Department of Physics and Materials Science, City University of Hong Kong, Tat Chee Avenue, Kowloon Tong, Hong Kong; ^4^ Clinical College of Integrated Traditional Chinese and Western Medicine, Anhui University of Chinese Medicine, Hefei, Anhui, China; ^5^ Collaborative Innovation Center of Radiation Medicine of Jiangsu Higher Education Institutions and School for Radiological and Interdisciplinary Sciences (RAD-X), Soochow University, Suzhou, Jiangsu, China; ^6^ Institute of Technical Biological & Agriculture Engineering, Hefei Institutes of Physical Sciences, Chinese Academy of Sciences, Hefei, Anhui, China; ^7^ School of Life Science, University of Science and Technology of China, Hefei, Anhui, China

**Keywords:** radiation-induced bystander effect, cytoplasm, Akt, mTOR, cytoplast

## Abstract

Cytoplasm is an important target for the radiation-induced bystander effect (RIBE). In the present work, the critical role of protein kinase B (Akt)/mammalian target of rapamycin (mTOR) pathway in the generation of RIBE signaling after X-ray irradiation and the rapid phosphorylation of Akt and mTOR was observed in the cytoplasm of irradiated human lung adenocarcinoma epithelial (A549) cells. Targeting A549 cytoplasts with individual protons from a microbeam showed that RIBE signal(s) mediated by the Akt/mTOR pathway were generated even in the absence of a cell nucleus. These results provide a new insight into the mechanisms driving the cytoplasmic response to irradiation and their impact on the production of RIBE signal(s).

## INTRODUCTION

The radiation-induced bystander effect (RIBE), an example of a non-targeted effect of ionizing radiation, occurs when signal(s) released by the irradiated cells to activate neighboring or distal non-irradiated cells and cause effects similar to direct irradiation, including the induction of chromosomal aberrations, changes of specific gene expression, genomic instability, mutation or even neoplastic transformation [1]. It is considered that RIBE can amplify the biological target area of radiation and cause genetic risks to the normal cells or tissues beyond the irradiated target in radiotherapy and other radiation exposures [1, 2]. Extensive studies on the health risks of radiation non-targeted effects, including RIBE, particularly in the low-dose regime have been carried out in recent decades [3, 4].

The roles of cellular cytoplasm and cytoplasmic organelles in radiobiological effect were revealed since Wu's findings [5], and further researches have investigated the important role of cytoplasm in RIBE. Shao *et al*. prove that both precise cytoplasmic and nuclear irradiation, with α-particle microbeam facility, induce distinct micronuclei (MN) increase in the co-cultured bystander cell population [6], and further study by Tartier *et al*. also show that cytoplasmic irradiation with the same facility induces 53BP1 protein relocalization in bystander cells though the peak of bystander 53BP1 foci induced by cytoplasmic irradiation is observed significantly later than that by nuclear irradiation [7]. Furthermore, the critical role of mitochondrial in cytoplasmic irradiation-induced bystander effect also has been revealed based on that the irradiated pseudo-ρ^0^ cells, which lack mitochondrial DNA, don't induce bystander effect but the irradiated cells with normal active mitochondrial function do [7–9]. It is speculated that the cytoplasm targeting causes mitochondrial activation and reactive oxygen species (ROS) production, then the signal(s) transduce into nucleus to induce the irradiated cells to secret RIBE signal(s) [7–9]. However, Byrne *et al*. suggest that the observed biological responses to cytoplasmic irradiation with microbeam X-rays or alpha particles could be attributed to a small amount of out-of-beam scattered ionizations in the nucleus [10]. As such, it is pertinent to carry out experiments with more unambiguous designs to study the RIBE induced by cytoplasm irradiation in order to better understand the mechanisms underlying the production and transduction of relevant RIBE signal(s).

In the present study, we found that the protein kinase B (Akt)/mammalian target of rapamycin (mTOR) pathway activated in the X-irradiated cytoplasm played an important role in the production of RIBE signal(s). Further studies, precisely targeting the cellular cytoplasm or enucleated cells (cytoplasts) with a proton microbeam, also showed that the Akt/mTOR pathway was critical in cytoplasm targeting-induced generation of RIBE signal(s) even in the absence of a nucleus, and this suggested that this signaling might be initiated and then play its role in RIBE in a *do novo* transcription independent manner.

## RESULTS

### Akt/mTOR mediating RIBE in transwell co-culture system

As regards the research on RIBE induced by X-ray, a transwell co-culture system was adopted [1, 2] and RIBE was assessed through the yield of micronucleus (MN) formation in bystander cells [6, 11]. Human lung adenocarcinoma epithelial A549 cells were employed for the present study. Those growing in each well (each with 1×10^6^ cells) of six-well plate were irradiated, while those cells growing in transwell inserts (1.0 μm pore size; Corning, Acton, MA, USA) were used as bystander cells. Before irradiation, 2 mL of fresh medium was replaced, and after irradiation the inserts were immediately put into each well and co-cultured for further analyses.

**Figure 1:**
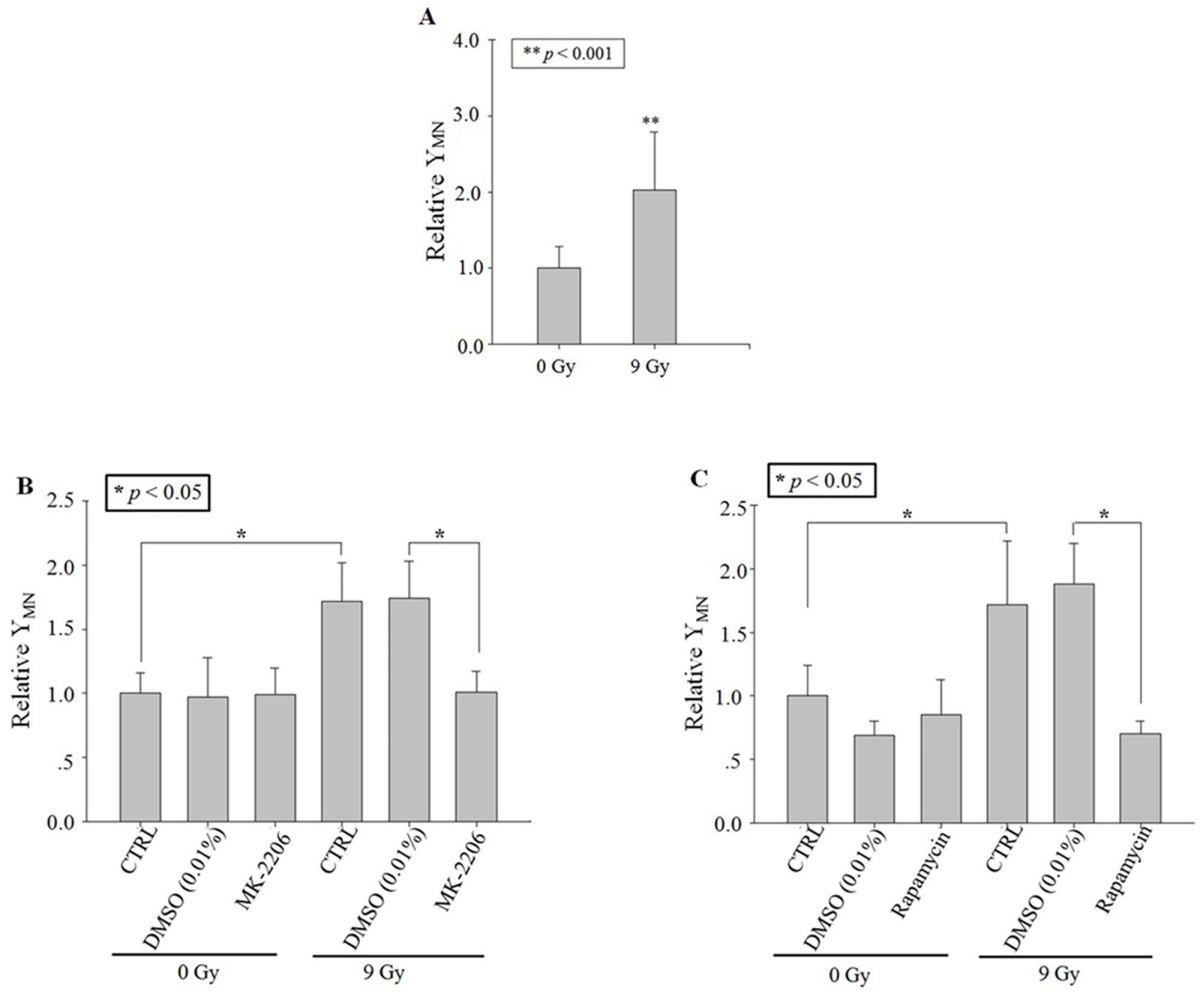
RIBE after X-ray irradiation Relative MN yields in bystander A549 cells co-cultured with cells irradiated with 9 Gy X-ray (in the transwell insert system). **A**. No drug treatment. **B**. Treating the irradiated cells with MK-2206 (an inhibitor of Akt). **C**. Treating the irradiated cells with Rapamycin (an inhibitor of m-TOR). Data were pooled from at least three independent experiments and the results are presented as means±S.D.

After 9 Gy X-ray irradiation, the yield of MN in bystander A549 cells increased distinctly to ~two folds of control (Figure 1A). To ensure the role of Akt and mTOR in the generation of RIBE, the specific inhibitors of Akt (MK-2206, 10 μmol/L; Sigma, St. Louis, MS, USA) and of mTOR (rapamycin, 200 nmol/L; Sigma, St. Louis, MS, USA) [12, 13] were used to treat only the irradiated cells but not the bystander cells for only 1 h before irradiation, and then removed from the well. Results in Figure 1B and 1C showed that the MN yields decreased significantly with either MK-2206 or rapamycin treatment respectively.

### Activation of Akt and mTOR in X-ray irradiated cells

**Figure 2:**
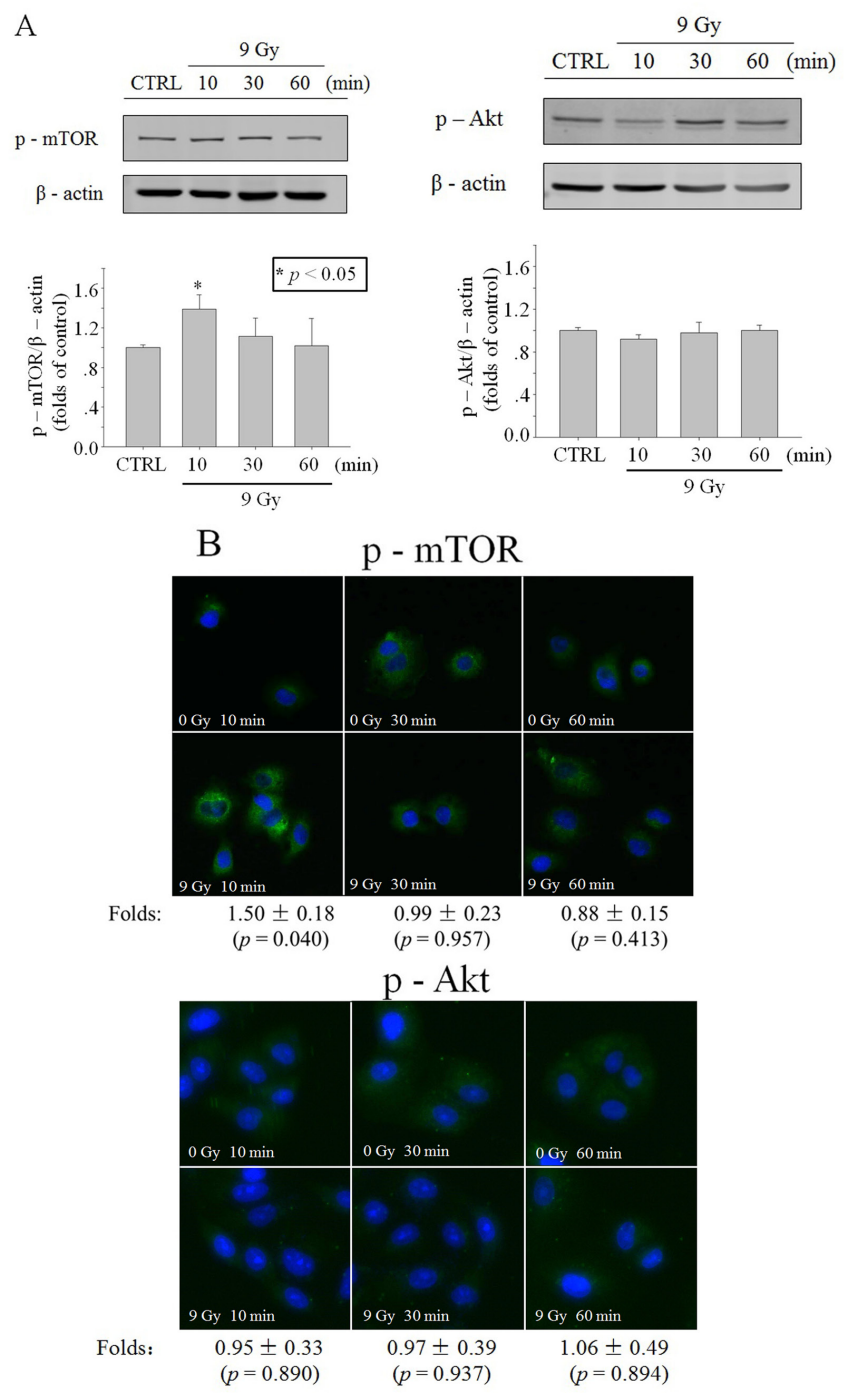
Activation of Akt/mTOR in whole cells after X-ray irradiation Time function of p-mTOR or p-Akt level in A549 cells irradiated with 9 Gy X-ray, revealed through western blot **A**. or immunofluorescence **B**. (blue: Hoechst; green: FITC). Data were pooled from at least three independent experiments and the results are presented as means±S.D.

**Figure 3:**
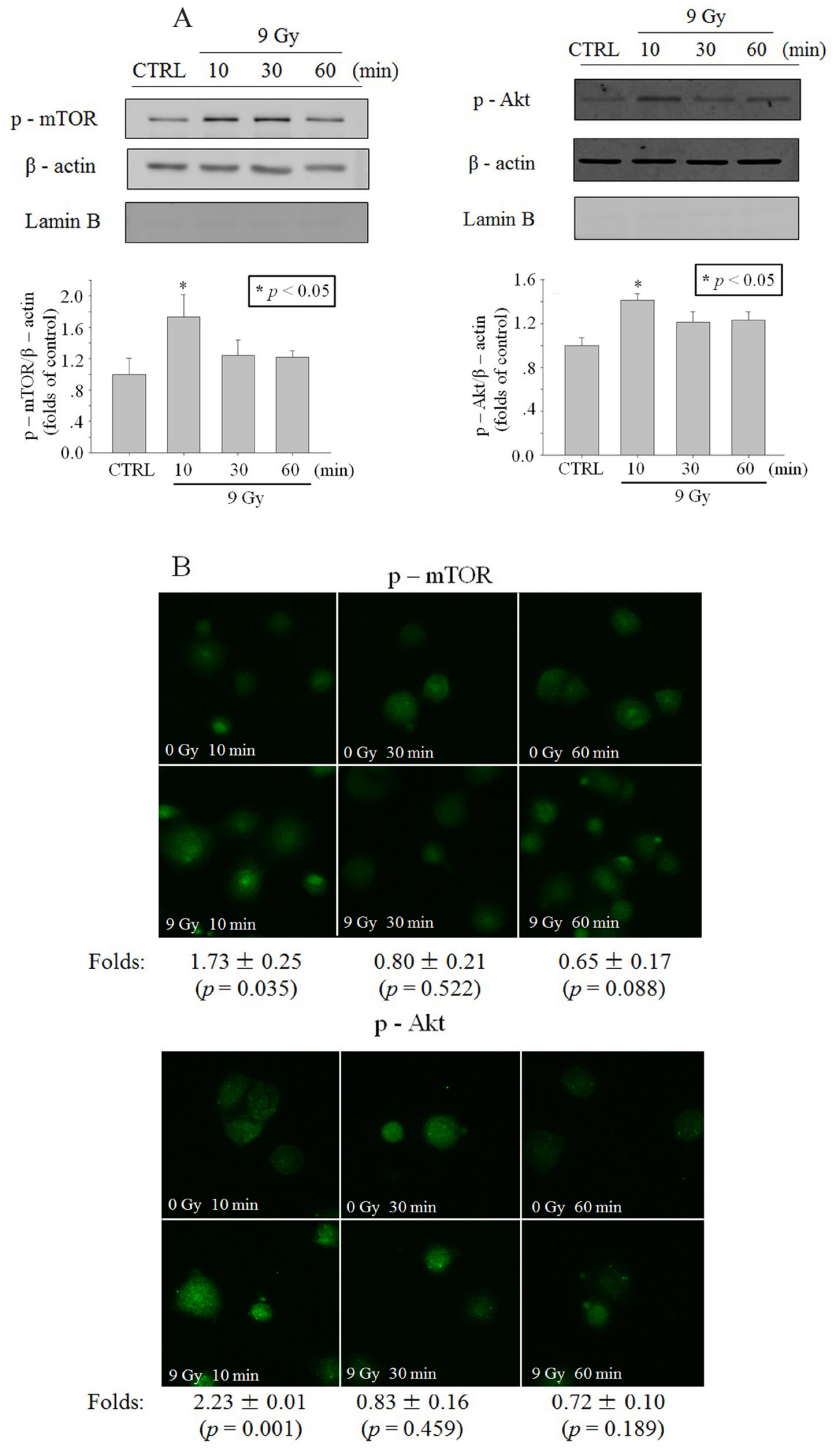
Activation of Akt/mTOR in cytoplasm after X-ray irradiation Time function of p-mTOR or p-Akt level after irradiation of 9 Gy X-ray. **A**. In A549 cell cytoplasm lysis revealed through western blot. **B**. In A549 cytoplasts revealed through immunofluorescence (blue: Hoechst; green: FITC). Data were pooled from at least three independent experiments and the results are presented as means±S.D.

To elucidate the activation of Akt and mTOR by the X-ray (9 Gy) irradiation, protein expression of mTOR and phosphorylated mTOR (Ser 2448) was detected with western blot and immunofluorescence. Results showed that X-ray (9 Gy) irradiation did not induce distinct change of mTOR protein expression in the whole cell lysis (Supplementary Figure 2A), but induced transient mTOR phosphorylation at 10 min post irradiation (Figure 2A). The protein expression levels of Akt, the upregulator of mTOR, and p-Akt (Thr 308) did not show distinct changes in the whole cell lysis (Supplementary Figure 2B; Figure 2A). The results of p-mTOR and p-Akt immunofluorescence detection also showed similar trends to those of western blot (Figure 2B and 3B).

Since the previous studies have shown that Akt is activated in the cytoplasm [14], we detected p-Akt level in cytoplasmic lysis and the results showed that p-Akt level elevated transiently at 10 min after irradiation (Figure 3A). To detect p-Akt with immunofluorescence, the enucleated A549 cells (cytoplasts) were made to avoid the influence of the nucleus. Results in Figure 3B also showed that a transiently phosphorylaton of Akt occurred at 10 min after irradiation. Similar to p-Akt, the level of p-mTOR also elevated transiently in cytoplasm at 10 min after irradiation (Figure 3A and 3B).

### Enucleated cytoplast irradiation induced RIBE

A549 cells were denucleated according to the methods described in ref. [15], and then the A549 cytoplasts were irradiated precisely with the microbeam facility at CAS-LIBB, which allowed individual protons to be delivered to cells with high reproducibility (single ion delivered with 99% efficiency) and high accuracy (99% within 5 μm) [16]. About 1,000 fluorescent A549 cells/cytoplasts were seeded in the central area (5 mm in diameter) of a specially designed microbeam dish consisting of a 3.5 μm-thick polypropylene film base (Collaborative Biomedical Products, Bedford, MA, USA). The non-fluorescent bystander cells were seeded in six individual circular areas (5 mm in diameter; ~1,000 cells in each area), which were evenly distributed around the central area (~8 mm apart) in the same microbeam dish. The pattern of irradiated and bystander cells in the microbeam dish was shown in Supplementary Figure 1.

**Figure 4:**
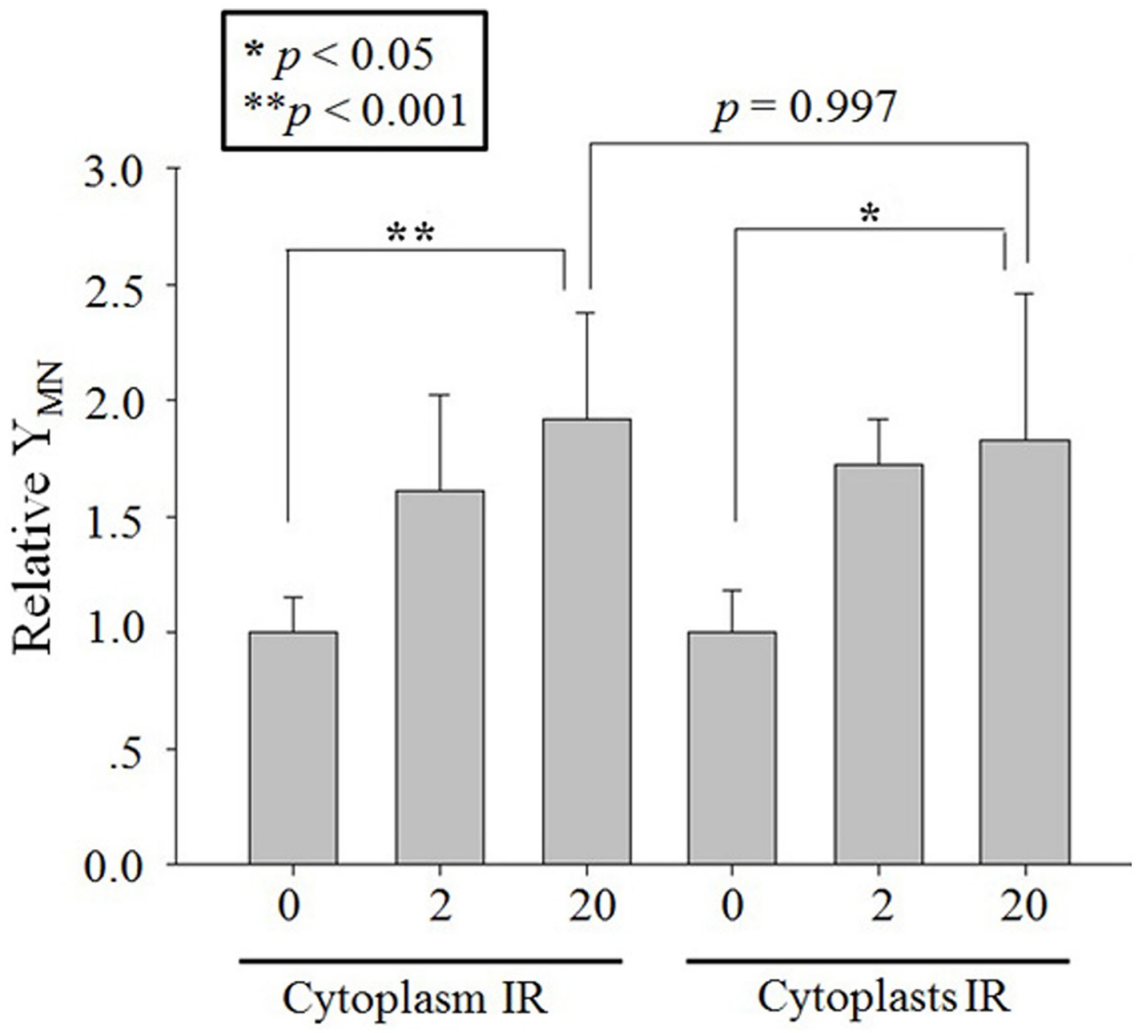
RIBE after proton targeting Relative MN yields in bystander A549 cells co-cultured with 2 or 20 protons targeted twenty A549 cytoplasts or cytoplasm part of twenty A549 cells. Data were pooled from at least four independent experiments and the results are presented as means±S.D.

Twenty A549 cytoplasts in the irradiated population were randomly selected and precisely targeted with 2 or 20 protons through the center of each cytoplast. The MN yield of the bystander cell population distinctly increased to 1.72 ± 0.19 folds (2 protons) and 1.83 ± 0.63 folds (20 protons) of the control (Figure 4). Similarly, 2 or 20 protons targeting the cytoplasm of twenty whole A549 cells, which were regarded as the positive control of cytoplast targeting, also increased the MN yield of bystander cell population significantly (Figure 4). No significance was observed between the RIBE intensity induced by cytoplasm- and cytoplast-targeting with 2 or 20 protons. These results indicated that targeting of cytoplasts, though the nucleus was absent, also induced distinct RIBE and its intensity was similar to that observed with cytoplasm targeting.

### Role of Akt/mTOR in cytoplasts targeting-induced RIBE

**Figure 5:**
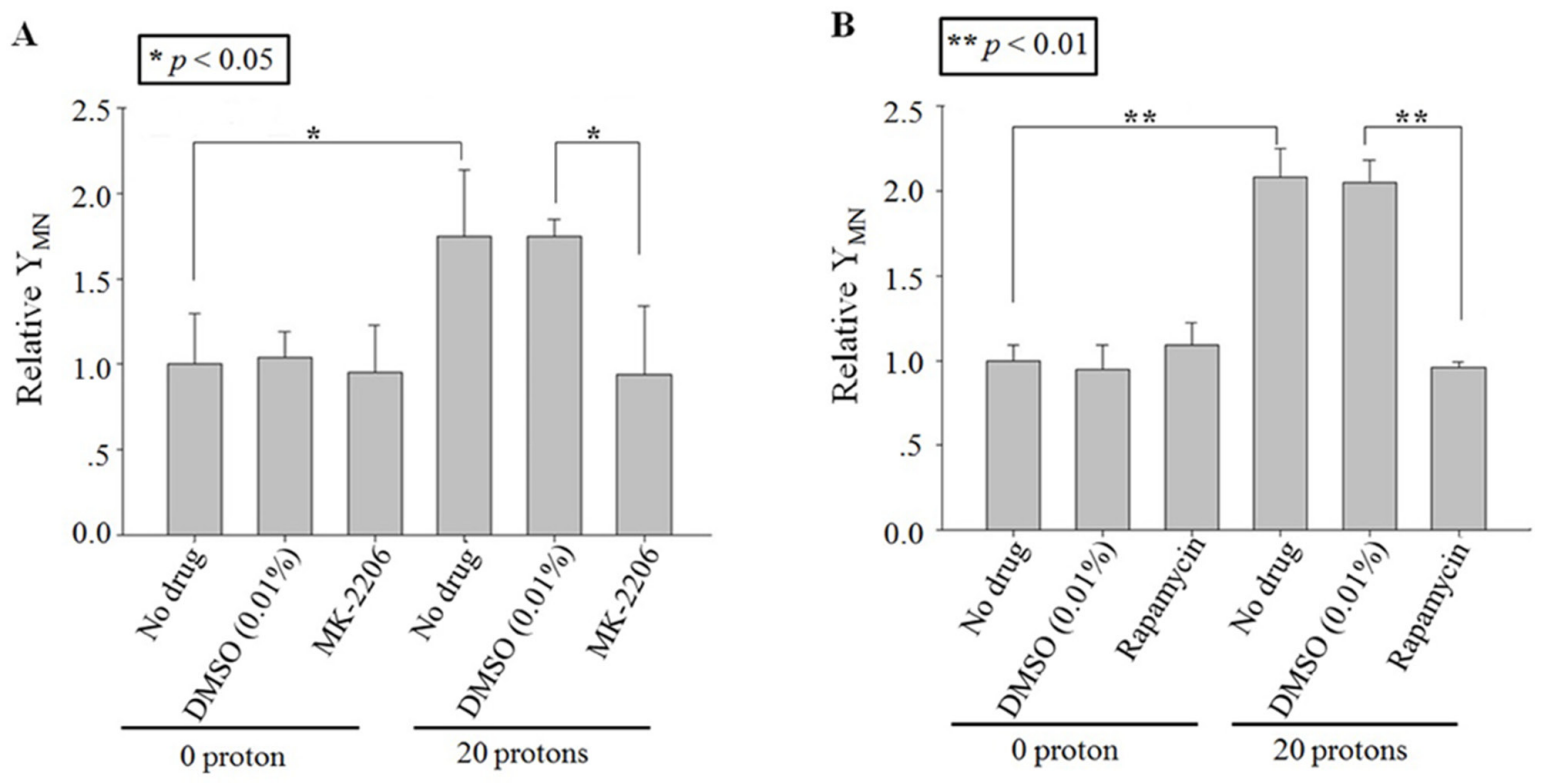
Effect of inhibitors on cytoplasts targeting-induced bystander effect Relative MN yields in bystander A549 cells co-cultured with 20 protons targeted twenty A549 cytoplasts, which were treated with MK-2206 **A**. or rapamycin **B**. before irradiation. Data were pooled from at least three independent experiments and the results are presented as means±S.D.

To ensure the role of Akt or mTOR in RIBE induced by cytoplasts targeting, the specific inhibitors, MK-2206 (Akt) or rapamycin (mTOR) [12, 13], were also used to treat only the irradiated population but not the bystander population at 1 h before irradiation and then removed. Twenty cytoplasts in the irradiated population were also chosen randomly and each of them was targeted with 20 protons. Results in Figure 5 showed that the MN yield decreased significantly to the background level with MK-2206 or Rapamycin treatment. These results suggested that the function of Akt/mTOR was also important in the production of RIBE signaling induced by cytoplasts targeting.

### Rapid activation of Akt/mTOR in a nucleus-independent manner

**Figure 6:**
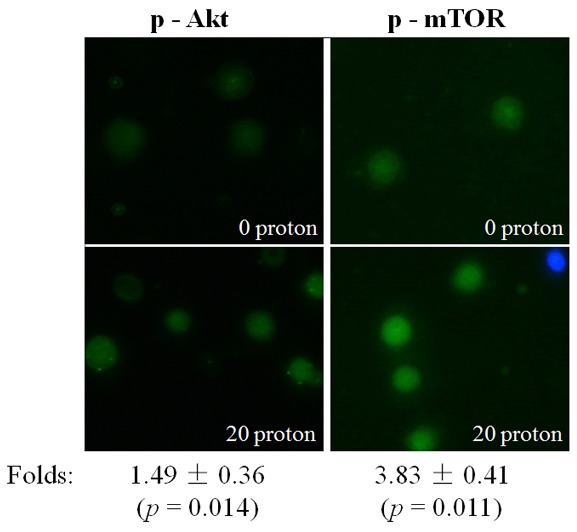
Activation of Akt/mTOR after proton targeting Expression of p-Akt or p-mTOR in A549 cytoplasts at 10 min after targeting by 20 protons, revealed through immunofluorescence (blue: Hoechst; green: FITC). Data were pooled from at least three independent experiments and the results are presented as means±S.D.

The phosphorylated proteins of Akt and mTOR in the targeted A549 cytoplasts were detected with immunofluorescence. Similar to the observations after X-ray irradiation, a significant increase of phosphorylated Akt and mTOR was detected 10 min after proton irradiation (Figure 6). These results suggested that proton irradiation could also effectively activate Akt/mTOR in the absence of a nucleus.

## DISCUSSION

The role of the cytoplasm in the generation of RIBE signal(s) has been investigated previously with a charged particle microbeam facility [6, 7]. No significant difference of RIBE intensity, assessed by MN or 53BP1 foci [6, 7], was observed between targeting the nucleus or cytoplasm only, but RIBE peak intensity occurs at a later time after cytoplasmic targeting [7]. Further mechanistic studies revealed that metabolically active mitochondrial function in directly irradiated cells plays an important role [7]. In this work, we investigated the activation and role of RIBE-related cytoplasmic signaling, and we observed that the irradiation-activated Akt/mTOR pathway played an important role in the production of RIBE. Our results (Figure 2, 3A) showed that inhibition of Akt/mTOR blocked X-ray irradiating whole cells-induced RIBE and the X-irradiation induced the activation of Akt or mTOR in the cytoplasm of irradiated whole cells with western blot/immunofluorescence. Further results of immunofluorescence (shown in Figure 3B) suggested that direct nucleus-independent activation of Akt/mTOR by irradiation was possible since a distinct fluorescence increase of p-Akt/p-mTOR was detected in the irradiated cytoplasts. Results of targeting cytoplasts with microbeam facility (Figure 4, 5, 6) further indicated that the activated Akt/mTOR in the cytoplasts was very critical to the production of RIBE signal(s).

Phosphorylation of Akt and mTOR were observed rapidly at 10 min post irradiation in both cytoplasm (X-ray irradiation) and enucleated A549 cells (proton targeting) (Figure 2, 3 and 6). Treating only the irradiated whole or enucleated cells with the inhibitors of Akt or mTOR significantly reduced the MN yield in bystander cells to the control levels (Figure 1 and 5), and these results indicated that the Akt/mTOR pathway in irradiated cells was critical to the generation of RIBE.

The Akt/mTOR pathway has been reported to be activated by radiation, and inhibition of this pathway is a novel radio-sensitizing therapeutic [17, 18]. Furthermore, Ivanov *et al*. have reported the activation of the PI3K-Akt pathway, stimulated by the IGF-1-Receptor kinase, in both directly irradiated (with an α-particle dose of 50 cGy) and bystander fibroblasts [19]. Rapid activation of Akt/mTOR in A549 cells by irradiation was reported by Chen *et al*. They reveal that 5 Gy X- irradiation induce rapid phosphorylation of Akt, peaking at 15 min post irradiation, in A549 cells and the phosphorylated level also decreases rapidly to background levels 60 min after irradiation, and a similar trend is also observed in radiation-induced phosphorylation of mTOR [20]. As well as ionizing radiation, it has also been reported that UVB induces rapid transient phosphorylation of Akt (peaking at 30 min after treatment) [21] and insulin treatment even induces transient phosphorylation more rapidly (peaking at 5 min after treatment and then dephosphorylated) [22].

Interestingly, Akt/mTOR-mediated RIBE signaling showed a nucleus independent manner in the present work. This was supported by the distinct increase of MN yield in bystander cells when cytoplasts were targeted, while the excessive MN yield was significantly suppressed by treating the targeted cytoplasts with inhibitors of Akt/mTOR (Figure 4 and 5). Nucleus-independent signaling has been reported previously in the apoptosis induced by cisplatin, which initiates cytoplasmic cdk2 to play a critical role in apoptosis in enucleated mouse kidney proximal tubule cells [23]. Althouth mTOR activation was consindered as a stress response to DNA damage [24], mTOR, a central regulator of cellular growth and metabolism, also has been reported to be involved in the nucleus-independent cytoplasmic signaling. Panaretakis *et al*. find that Interferon alpha-induced apoptosis, *via* Bak and Bax and the mitochondrial pathway, is mediated through activation of PI3K/mTOR, extracellular signal-regulated kinase (ERK) and c-Jun NH2-terminal kinase (JNK) in a nucleus-independent manner based on the results from enucleated RHEK-1 cells [25]. Marques *et al*. also showed that when skeletal muscle and neural progenitor cells ceased dividing and progressd in the cytoplast but not in the nucleus [26]. Our present results indicated that the cytoplasmic signal pathway(s), which were initiated by irradiation in *de novo* transcription manner, was involved in the generation of RIBE signal(s).

Radiation-stimulated production of reactive oxygen species (ROS) are also regarded as essential activators in the generation of RIBE signals [6, 27]. Scavenging ROS inhibiting bystander effect effectively has been reported in many previous studies [1, 2, 28]. Lower production of ROS after irradiation in mitochondrial DNA lacking ρ^0^ cells compared with the normal cells is considered as one main reason by which ρ^0^ cells irradiation doesn't induce a bystander effect [7–9]. In the present work, with the fluorescent probe, we detected that the ROS production continued to increase within 1 h after proton targeting of A549 cytoplasts even although the nucleus was absent (Supplementary Figure 3). Previous studies have reported that ROS production stimulates or mediates the activation of Akt/mTOR signaling in response to some stimuli [29–31]. Other studies show that mTOR is necessary in regulating mitochrondiral functions such as oxygen consumption and oxidative capacity [32, 33] *via* targeting transcription factor yin-yang 1 [34]. Moreover, signaling of the cell membrane, a more sensitive target of radiation than cytoplasm, also has been proved to be involved in RIBE transduction [6, 7, 35]. Bowers *et al*. report that radiation activate epidermal growth factor (ERBB) family receptors in the plasma membrane within 5-10 min post irradiation [36], and ERBB receptors have been reported to cause activation of Akt pathways *via* enhancing the activities of RAS family transducer molecules [37]. It is well known that Akt is phosphorylated by translocating and binding with the membrane proteins PtdIns(3,4,5)P3 and PtdIns(3,4)P2, which are produced by activated PI3K [38]. So further studies should be performed to explore how proton targeting initiated the Akt/mTOR activation and how about the relationship between Akt/mTOR and the reported RIBE signaling.

As one manifestation of the non-targeted effects of radiation, RIBE is considered to potentially increase the risk of radiation especially in the low-dose range. RIBE induced by cytoplasmic targeting with a microbeam confirms that the cytoplasm is also an important target for genotoxic effects of ionizing radiation. The risk of cytoplasmic traversal needs to be assessed as the possibility of cytoplasmic only traversal is higher than nuclear traversal after low dose radiation especially environmental radon exposure [5–7, 39–42]. Although our study demonstrated the existence of radiation activating Akt/mTOR pathway in nucleus independent manner to mediate the production of RIBE signal(s), the limited results could not clarify the relationship between the nucleus-independent pathway(s) and nucleus associated pathway(s) of RIBE. Further studies are needed to explore the complex signaling pathways and organelles involved in the cytoplasmic traversal-induced bystander effect.

## MATERIALS AND METHODS

### Cell culture

A549 cells, obtained from Cell Bank, Type Culture Collection, Chinese Academy of Sciences (CBTCCCAS), were cultured in high glucose DMEM medium (Thermo Scientific Hyclone, Logan, UT, US) supplemented with 10% heat-inactivated fetal bovine serum (FBS; Thermo Scientific Hyclone, Logan, UT, US), 10 mg/mL streptomycin (Sigma, St. Louis, MS, USA), and 100 IU/mL penicillin (Sigma, St. Louis, MS, USA). The cells were maintained in a humidified incubator under 5% CO_2_ at 37°C. The cell line was authenticated by STR profile analysis and routinely checked for contamination by mycoplasma using Hoechst staining and consistently found to be negatively.

### Enucleation of A549 cells

Briefly, after trypsinization, the A549 cell suspension was centrifuged at 1000 rpm for 5 min. The harvested cells were resuspended with the culture medium containing cytochalasin B (Sigma, St. Louis, MS, USA) at a concentration of 10 μg/mL and then incubated for 30 min at 37°C. After this, the cells were resuspended with 1 ml cooling DMEM medium with 50% Percoll (Sigma, St. Louis, MS, USA) and the cell suspension was isopycnic gradiently centrifuged at 14000 rpm for 70 min at 34°C. The cytoplasts made from A549 cells were purified by resuspending with cooling DMEM medium and centrifuged at 3000 rpm for 5 min at 10 °C twice. The final enucleated A549 cells were resuspended with fresh culture medium and identified by staining with Hoechst 33342 (10 μg/mL; Invitrogen, Eugene, OR, USA) and CellTracker orange CMRA (5 μmol/L; Life Technologies, Eugene, OR, USA) for 30 min at 37°C. With this method, the fraction of cytoplasts was about 60%. The viability of the cytoplasts was assessed with FDA (10 ng/mL; Sigma, St. Louis, MS, USA). The fluorescent images were captured with a fluorescent microscope (Leica DMI 4000B; Wetzlar, German).

### X-ray irradiation

The broad-beam irradiation was performed with a 120 kVp X-ray machine (Kangmeng XSZ-220/20; Dandong, China) at dose rate of 2.02 Gy/min (120 kV, 12.2 mA).

### Microbeam irradiation

The microbeam facility at CAS-LIBB was used for this study [16]. The irradiated cells/cytoplasts were stained with Hoechst 33342 (10 μg/mL; Invitrogen, Eugene, OR, USA) and CellTracker orange CMRA (5 μmol/L; Life Technologies, Eugene, OR, USA), and the bystander cells were not stained. About 1,000 fluorescent A549 cells/cytoplasts were seeded in the central area (5 mm in diameter) of a specially designed microbeam dish consisting of a 3.5 μm-thick polypropylene film base (Collaborative Biomedical Products, Bedford, MA, USA) (Supplementary Figure 1). The regions prepared for cell seeding had been pretreated with 3.5 μg/cm^2^ Cell-Tak adhesive (BD, Bedford, MA, USA) [1, 2].

The positions of each CellTracker orange-stained cell cytoplasm or Hoechst-stained nucleus were found by using the computerized imaging system of CAS-LIBB microbeam facility [16], and their coordinates were stored to be revisited and irradiated automatically. For cytoplasmic irradiation of whole A549 cells, 20 randomly selected cells within the irradiated population were traversed individually at a single location through their cytoplasm with 2 or 20 protons (3.0 MeV) at least 5 μm from the nucleus [16]. Alternatively, for enucleated cells irradiation, the randomly selected 20 cytoplasts were individually traversed with 2 or 20 protons through the center of each cytoplast. The irradiation was performed at ~2 h after cytoplasts attaching on the polypropylene film.

In some experiments, the chemical inhibitors were used to treated the cytoplasts in the central area for 1 h, washing with basic medium for three times, and then the bystander cells were seeded in the bystander areas of microbeam dish.

### MN scoring

The cytokinesis block technique was used to assay MN *in situ* [11][Fenech, 2000 #486]. At 1 h after irradiation, cytochalasin B (Sigma, St. Louis, MS, USA) was added into the culture medium at a final concentration of 1.5 μg/mL, and then the cells were incubated for a further 48 h. After incubation, the cells were fixed with 2% paraformaldehyde for 30 min and stained with acridine orange (10 μg/mL, Sigma, St. Louis, MS, USA). MN were scored in the binucleated cells and classified according to standard criteria under a fluorescent microscope (Leica DMI 4000B; Wetzlar, German). The MN frequency was calculated as the ratio of the number of MN to the scored number of binucleated cells [11]. At least 1000 binucleated cells were counted.

### Western blot

Total protein was extracted with RIPA (Beyotime Biotechnology, Shanghai, China) and the concentration was determined by a BCA protein assay kit (Beyotime Biotechnology, Shanghai, China). The nuclear and cytosolic proteins were extracted separately with a nuclear and cytoplasmic protein extraction kit (Beyotime Biotechnology, Shanghai, China) according to the manufacturer's protocols. Equal amounts of proteins (100 μg) were fractionated by 8% or 10% SDS-PAGE and transferred onto PVDF membranes (Millipore, Billerica, MA, USA). The membranes were blotted with the primary antibodies rabbit monoclonal anti-human p-Akt (Thr 308; 1:1000; catalog No. 2965, clone No. C31E5E, Cell Signaling, Boston, MA, USA), mouse monoclonal anti-human Akt (1:1000; catalog No. 2920, clone No. 40D4, Cell Signaling, Boston, MA, USA), rabbit monoclonal anti-human p-mTOR (Ser 2448; 1:1000; catalog No. 109268, clone No. EPR426(2), Abcam, Cambridge, UK), mouse monoclonal anti-human mTOR (1:1000; catalog No. 4517, clone No. L27D4, Cell Signaling, Boston, MA, USA), and mouse monoclonal anti-human β-actin (1:5000; catalog No. 3700, clone No.8H10D10, Cell Signaling, Boston, MA, USA) and mouse monoclonal anti-human Lamin B (1: 500; catalog No. 374015, clone No.D0324 Santa Cruz, Paso Robles, CA, USA), and then with the fluorescent secondary antibodies (Alex Fluor® 790 goat anti-rabbit IgG, 1:10,000, Catalog No. 111-655-144; Alex Fluor® 680 goat anti-mouse IgG, 1:10,000, Catalog No. 115-625-146; LI-COR, Lincoln, NE, USA). The membranes were detected and analyzed with an Odyssey^®^ CLx Infrared Imaging System (LI-COR, Lincoln, NE, USA).

### Immunofluorescence

After fixing with methanol/acetone [3:7 (v/v)] for 5 min at −20 °C and washing with PBS for three times, the cells were blocked with PBS^+^solution(PBS supplemented with 1% BSA) for 30 min at room temperature. The cells were then incubated with rabbit anti-p-Akt (Thr 308; 1:1600; catalog No. 2965, clone No. C31E5E, Cell Signaling, Boston, MA, USA) or rabbit anti-p-mTOR (Ser 2448; 1:100; catalog No. 109268, clone No. EPR426(2), Abcam, Cambridge, UK) at 4°C overnight, washed with PBST (PBS supplemented with 0.05% Tween-20) for 3×15 min, and then incubated in PBS^+^ containing secondary goat anti-rabbit IgG-FITC (1:100; catalog No. sc-2012, Santa Cruz, Paso Robles, CA, USA,) for a further 60 min at room temperature. After another wash with PBST for 3×15 min, the cells were counterstained with Hoechst 33342 (10 μg/mL; Invitrogen, Eugene, OR, USA) for 20 min at room temperature. After a final wash with PBST, the samples were mounted with a drop of mounting medium (BioRad, Hercules, CA, USA), sealed with nail polish, and the fluorescent images of at least 100 cells were captured with a fluorescent microscope (Leica DMI 4000B; Wetzlar, German). The intensity of fluorescence was analyzed with the free software Image J.

### Statistical analysis

Statistical analysis was performed on the data obtained from at least three independent experiments. The data were presented as means ± SD. The significance was accessed by using Student's *t*-test. A *p*-value less than 0.05 between two independent groups was considered significant.

## SUPPLEMENTARY MATERIALS FIGURES



## Abbreviations

RIBE, radiation-induced bystander effect; mTOR, mammalian target of rapamycin; Akt, proteinkinase B; MN, micronuclei; ROS, reactive oxygen species; ERK, extracellular signal-regulated kinase; JNK, c-Jun NH2-terminal kinase.

## References

[1] Prise KM, O’Sullivan JM (2009). Radiation-induced bystander signalling in cancer therapy. Nat Rev Cancer.

[2] Morgan WF, Sowa MB (2015). Non-targeted effects induced by ionizing radiation: Mechanisms and potential impact on radiation induced health effects. Cancer Lett.

[3] Leuraud K, Richardson DB, Cardis E, Daniels RD, Gillies M, O’Hagan JA, Hamra GB, Haylock R, Laurier D, Moissonnier M, Schubauer-Berigan MK, Thierry-Chef I, Kesminiene A (2015). Ionising radiation and risk of death from leukaemia and lymphoma in radiation-monitored workers (INWORKS): an international cohort study. Lancet Haematol.

[4] Little MP, Wakeford R, Tawn EJ, Bouffler SD, Berrington de Gonzalez A (2009). Risks associated with low doses and low dose rates of ionizing radiation: why linearity may be (almost) the best we can do. Radiology.

[5] Wu LJ, Randers-Pehrson G, Xu A, Waldren CA, Geard CR, Yu Z, Hei TK (1999). Targeted cytoplasmic irradiation with alpha particles induces mutations in mammalian cells. Proc Natl Acad Sci USA.

[6] Shao C, Folkard M, Michael BD, Prise KM (2004). Targeted cytoplasmic irradiation induces bystander responses. Proc Natl Acad Sci USA.

[7] Tartier L, Gilchrist S, Burdak-Rothkamm S, Folkard M, Prise KM (2007). Cytoplasmic irradiation induces mitochondrial-dependent 53BP1 protein relocalization in irradiated and bystander cells. Cancer Res.

[8] Chen S, Zhao Y, Han W, Zhao G, Zhu L, Wang J, Bao L, Jiang E, Xu A, Hei TK, Yu Z, Wu L (2008). Mitochondria-dependent signalling pathway are involved in the early process of radiation-induced bystander effects. Br J Cancer.

[9] Zhou H, Ivanov VN, Lien YC, Davidson M, Hei TK (2008). Mitochondrial function and nuclear factor-κB-mediated signaling in radiation-induced bystander effects. Cancer Res.

[10] Byrne HL, Domanova W, McNamara AL, Incerti S, Kuncic Z (2015). The cytoplasm as a radiation target: an in silico study of microbeam cell irradiation. Phys Med Biol.

[11] Fenech M (2000). The in vitro micronucleus technique. Mutat Res.

[12] Cheng Y, Ren X, Zhang Y, Patel R, Sharma A, Wu H, Robertson GP, Yan L, Rubin E, Yang JM (2011). eEF-2 kinase dictates cross-talk between autophagy and apoptosis induced by Akt Inhibition, thereby modulating cytotoxicity of novel Akt inhibitor MK-2206. Cancer Res.

[13] Wu MY, Fu J, Xu J, O’Malley BW, Wu RC (2012). Steroid receptor coactivator 3 regulates autophagy in breast cancer cells through macrophage migration inhibitory factor. Cell Res.

[14] Du K, Tsichlis PN (2005). Regulation of the Akt kinase by interacting proteins. Oncogene.

[15] Gudas JM, Karenlampi SO, Hankinson O (1986). Intracellular location of the Ah receptor. J Cell Physiol.

[16] Wang X, Wang X, Chen L, Hu Z, Li J, Wu Y, Wu L, Yu Z (2004). Development of CAS-LIBB single-particle microbeam for localized irradiation of living cells. Chinese Science Bulletin.

[17] Chang L, Graham PH, Hao J, Ni J, Bucci J, Cozzi PJ, Kearsley JH, Li Y (2014). PI3K/Akt/mTOR pathway inhibitors enhance radiosensitivity in radioresistant prostate cancer cells through inducing apoptosis, reducing autophagy, suppressing NHEJ and HR repair pathways. Cell Death Dis.

[18] Albert JM, Kim KW, Cao C, Lu B (2006). Targeting the Akt/mammalian target of rapamycin pathway for radiosensitization of breast cancer. Mol Cancer Ther.

[19] Ivanov VN, Zhou H, Ghandhi SA, Karasic TB, Yaghoubian B, Amundson SA, Hei TK (2010). Radiation-induced bystander signaling pathways in human fibroblasts: a role for interleukin-33 in the signal transmission. Cell Signal.

[20] Chen YH, Pan SL, Wang JC, Kuo SH, Cheng JC, Teng CM (2014). Radiation-induced VEGF-C expression and endothelial cell proliferation in lung cancer. Strahlenther Onkol.

[21] Nomura M, Kaji A, Ma WY, Zhong S, Liu G, Bowden GT, Miyamoto KI, Dong Z (2001). Mitogen- and Stress-activated Protein Kinase 1 Mediates Activation of Akt by Ultraviolet B Irradiation. J Biol Chem.

[22] Bruss MD, Arias EB, Lienhard GE, Cartee GD (2005). Increased phosphorylation of Akt substrate of 160 kDa (AS160) in rat skeletal muscle in response to insulin or contractile activity. Diabetes.

[23] Yu F, Megyesi J, Price PM (2008). Cytoplasmic initiation of cisplatin cytotoxicity. Am J Physiol Renal Physiol.

[24] Proud CG (2004). The multifaceted role of mTOR in cellular stress responses. DNA Repair.

[25] Panaretakis T, Hjortsberg L, Tamm KP, Björklund AC, Joseph B, Grandér D (2008). Interferon alpha induces nucleus-independent apoptosis by activating extracellular signal-regulated kinase 1/2 and c-Jun NH2-terminal kinase downstream of phosphatidylinositol 3-kinase and mammalian target of rapamycin. Mol Biol Cell.

[26] Marques L (2013). Dynamics of Akt activation during mouse embryo development: distinct subcellular patterns distinguish proliferating versus differentiating cells. Differentiation.

[27] Mikkelsen RB, Wardman P (2003). Biological chemistry of reactive oxygen and nitrogen and radiation-induced signal transduction mechanisms. Oncogene.

[28] Glebova K, Veiko N, Kostyuk S, Izhevskaya V, Baranova A (2015). Oxidized extracellular DNA as a stress signal that may modify response to anticancer therapy. Cancer Lett.

[29] Yang J, Li TZ, Xu GH, Luo BB, Chen YX, Zhang T (2013). Low-concentration capsaicin promotes colorectal cancer metastasis by triggering ROS production and modulating Akt/mTOR and STAT-3 pathways. Neoplasma.

[30] Hambright HG, Meng P, Kumar AP, Ghosh R (2015). Inhibition of PI3K/AKT/mTOR axis disrupts oxidative stress-mediated survival of melanoma cells. Oncotarget.

[31] Silva A, Gírio A, Cebola I, Santos CI, Antunes F, Barata JT (2011). Intracellular reactive oxygen species are essential for PI3K/Akt/mTOR-dependent IL-7-mediated viability of T-cell acute lymphoblastic leukemia cells. Leukemia.

[32] Ramanathan A, Schreiber SL (2009). Direct control of mitochondrial function by mTOR. Proc Natl Acad Sci USA.

[33] Morita M, Gravel SP, Hulea L, Larsson O, Pollak M, St-Pierre J, Topisirovic I (2015). mTOR coordinates protein synthesis, mitochondrial activity and proliferation. Cell Cycle.

[34] Cunningham JT, Rodgers JT, Arlow DH, Vazquez F, Mootha VK, Puigserver P (2007). mTOR controls mitochondrial oxidative function through a YY1–PGC-1α transcriptional complex. Nature.

[35] Nagasawa H, Cremesti A, Kolesnick R, Fuks Z, Little JB (2002). Involvement of membrane signaling in the bystander effect in irradiated cells. Cancer Res.

[36] Bowers G, Reardon D, Hewitt T, Dent P, Mikkelsen RB, Valerie K, Lammering G, Amir C, Schmidt-Ullrich RK (2001). The relative role of ErbB1-4 receptor tyrosine kinases in radiation signal transduction responses of human carcinoma cells. Oncogene.

[37] Lammering G, Hewit TH, Valerie K, Contessa JN, Amorino GP, Dent P, Schmidt-Ullrich RK (2003). EGFRvIII-mediated radioresistance through a strong cytoprotective response. Oncogene.

[38] Liu P, Cheng H, Roberts TM, Zhao JJ (2009). Targeting the phosphoinositide 3-kinase pathway in cancer. Nat Rev Drug Discov.

[39] Hong M, Xu A, Zhou H, Wu L, Randers-Pehrson G, Santella RM, Yu Z, Hei TK (2010). Mechanism of genotoxicity induced by targeted cytoplasmic irradiation. Br J Cancer.

[40] Nikezic D, Yu KN (2001). Alpha hit frequency due to radon decay products in human lung cells. International Journal of Radiation Biology.

[41] Nikezic D, Yu KN (2002). Distributions of specific energy in sensitive layers of human respiratory tract. Radiation Research.

[42] Nikezic D, Yu KN (2002). Alpha particle lineal energy spectra for the human lung. International Journal of Radiation Biology.

