# Supplementary material for: Akt/mTOR mediated induction of bystander effect signaling in a nucleus independent manner in irradiated human lung adenocarcinoma epithelial cells
Source: Oncotarget. 2017 Feb 1;8(11):18010–20. doi: 10.18632/oncotarget.14931 (PMC5392303; doi:10.18632/oncotarget.14931)
Supplement: Supplementary file 1 [file oncotarget-08-18010-s001.pdf]

Akt/mTOR mediated induction of bystander effect signaling in a nucleus independent manner in irradiated human lung adenocarcinoma epithelial cells

SUPPLEMENTARY FIGURES

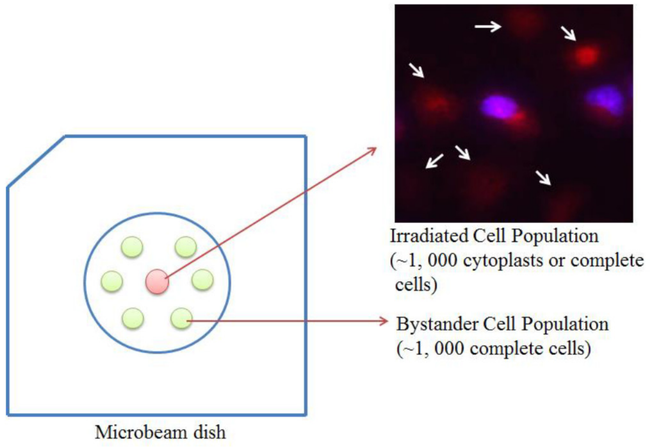

Supplementary Figure 1: Schematic representation of irradiated and bystander cell population in the microbeam dish. (White arrows: cytoplasts stained with CellTracker orange CMRA; Blue: Hoechst 33342).

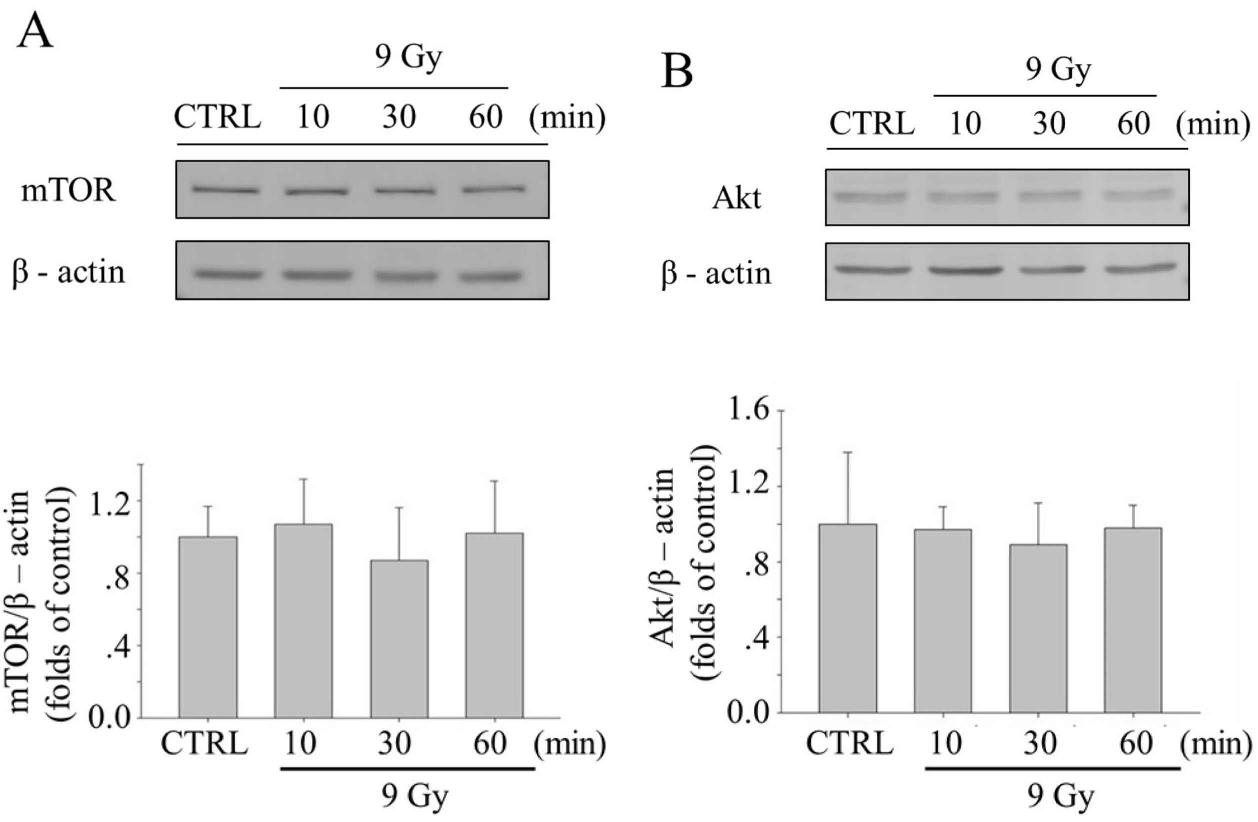

Supplementary Figure 2: Protein level in A549 cells irradiated with 9 Gy X-ray. A. mTOR. **B.** Akt. Data were pooled from three independent experiments and the results are presented as means ± S.D.

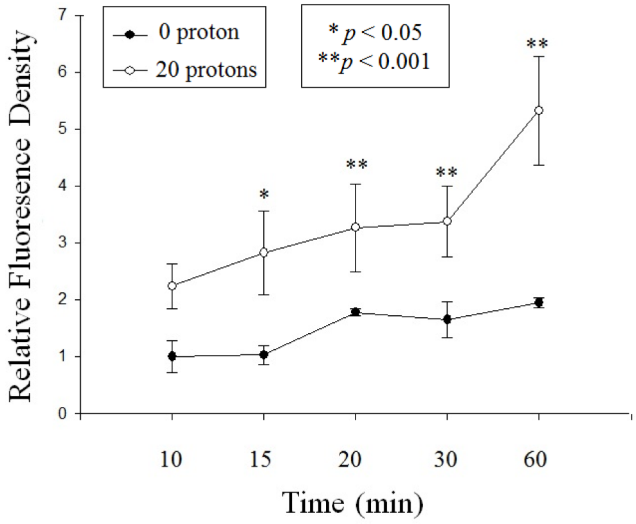

Supplementary Figure 3: ROS generation in proton targeted A549 cytoplasts by detecting with ROS fluorescent probe, 5-(and-6)-carboxy-2', 7′-dichlorodihydrofluorescein diacetate (carboxy-H2DCFDA). The cytoplasts were labelled with the probe just before irradiation and the fluorescence intensities of at least 100 cytoplasts were analyzed with Image J in each independent experiment. Data were pooled from at least three independent experiments and the results are presented as means ± S.D. Significance was calclulated between the irradiated and non-irradiated samples at the same timepoint.
